# A Literature Review of the Morphological Variability in the Intrinsic Muscles of the Foot: Traps Awaiting Clinicians during Ultrasound

**DOI:** 10.3390/jcm13154286

**Published:** 2024-07-23

**Authors:** Marta Pośnik, Nicol Zielinska, Bartosz Gonera, Łukasz Olewnik, Mariola Głowacka, Krystian Maślanka, Kacper Ruzik

**Affiliations:** 1 Department of Anatomical Dissection and Donation, Medical University of Lodz, 90-419 Łódź, Poland; marta.posnik@stud.umed.lodz.pl (M.P.); krystian.maslanka@stud.umed.lodz.pl (K.M.); 2 Department of Clinical Anatomy, Masovian Academy in Płock, 09-402 Płock, Poland; nicol.zielinska@stud.umed.lodz.pl (N.Z.); bartosz.gonera@umed.lodz.pl (B.G.); kacper.ruzik@umed.lodz.pl (K.R.); 3 Nursing Department, Masovian Academy in Płock, 09-402 Płock, Poland; m.glowacka@mazowiecka.edu.pl

**Keywords:** anatomical variations, imaging study, intrinsic muscles of the foot, morphological variability, ultrasound, ultrasound traps

## Abstract

**Purpose:** Like other muscular compartments of the human body, the intrinsic muscles of the foot present considerable morphological variability. The aim of this review was to present variations that can potentially cause problems during surgery but might be detected during an ultrasound examination. **Materials and methods:** PubMed was searched for relevant articles. The identified papers were listed, and citation tracking was performed. **Results:** Even though lower limb structure is well studied, the variations associated with the intrinsic muscles of the foot and their related ultrasound examination are not. **Conclusions:** The muscles and tendons of the foot demonstrate similar degrees of variance as other regions of the human body; however, this subject is not as widely covered in the literature. Further ultrasound studies are needed to build awareness of morphological variability in this region, as the findings could prevent misdiagnosis.

## 1. Introduction

The morphology of the human foot is markedly different from those of other species, not least due to our evolutional adaptation to bipedal posture [1]. The intrinsic muscles of the foot play a key role in maintaining this posture by acting as foot stabilizers; these are necessary because of the greater fluctuations in the center of gravity over the foot while balancing [2]. Nevertheless, the muscles present considerable variability regarding additional muscular heads, tendinous slips, additional muscular and tendinous connections among the components of muscular compartments, and the positions of the origins, aspects of insertions, or even the occurrence of accessory muscles [1,3,4,5]. It cannot be ruled out that such variation may indicate that the intrinsic muscles of the foot take part in an ongoing process of adaptation to maintain a bipedal posture [1]. 

Many of these variations may be important in situations when they are the main cause of pain and may also be useful in surgical procedures [6,7,8]. 

Ultrasound (US) imaging can reveal morphological variations in the muscles from different areas of the human body [9,10,11,12,13], and the foot region seems indifferent. Moreover, this method of visualization is increasingly used in the planning and preparation of surgical procedures in the foot region [14,15,16]. However, to avoid misdiagnosis that can potentially affect surgery, the clinician working in this area needs to be aware of the potential variations in foot anatomy.

The aim of this literature review is to present common variations in the anatomy of intrinsic foot muscles that may potentially mislead clinicians during ultrasound imaging for the diagnosis of various symptoms reported in the foot region. 

## 2. Review Design and Methods

An electronic search in the “PubMed” database was performed to find suitable publications for the presented review of the literature. 

The following terms were used during the search strategies: extensor digitorum brevis: anatomical variation, morphological variability, classification, muscle variation, ultrasound visualization, ultrasound study, imaging study; extensor hallucis brevis: anatomical variation, muscle variation, morphological variability, classification, ultrasound visualization, ultrasound study, muscle variation, imaging study; abductor hallucis: anatomical variation, muscle variation, morphological variability, classification, ultrasound visualization, ultrasound study, imaging study; adductor hallucis: muscle variation, anatomical variation, morphological variability, classification, ultrasound visualization, ultrasound study, imaging study; flexor hallucis brevis: muscle variation, anatomical variation, morphological variability, classification, ultrasound visualization, ultrasound study, imaging study; flexor digitorum brevis: muscle variation, anatomical variation, morphological variability, classification, ultrasound visualization, ultrasound study, imaging study; quadratus plantae: muscle variation, anatomical variation, morphological variability, classification, ultrasound visualization, ultrasound study, imaging study; lumbrical muscles of foot: anatomical variation, muscle variation, morphological variability, classification, ultrasound visualization, ultrasound study, imaging study; plantar interossei muscles: anatomical variation, muscle variation, morphological variability, classification, imaging study, ultrasound study, ultrasound visualization; abductor digiti minimi muscle: anatomical variation, muscle variation, morphological variability, classification, ultrasound visualization, ultrasound study, imaging study; flexor digiti minimi brevis: muscle variation, anatomical variation, morphological variability, classification, ultrasound visualization, ultrasound study, imaging study; and opponens digiti minimi: anatomical variation, muscle variation, morphological variability, classification, ultrasound visualization, ultrasound study, imaging study, ultrasound visualization of the muscles. 

The exclusion criteria included the following:

- Manuscript written in any language other than English.
- Lack of information regarding the description of the typical anatomy/morphological variability in a structure covered by the topic of this review/clinical significance/application of imaging studies/the use of US in diagnosis.
- Articles focused mainly on methods of visualization other than US imaging.
- Type of the article including expert opinion/letter to the editor/conference report.
- Publication after March 2024.

**Figure 1:**
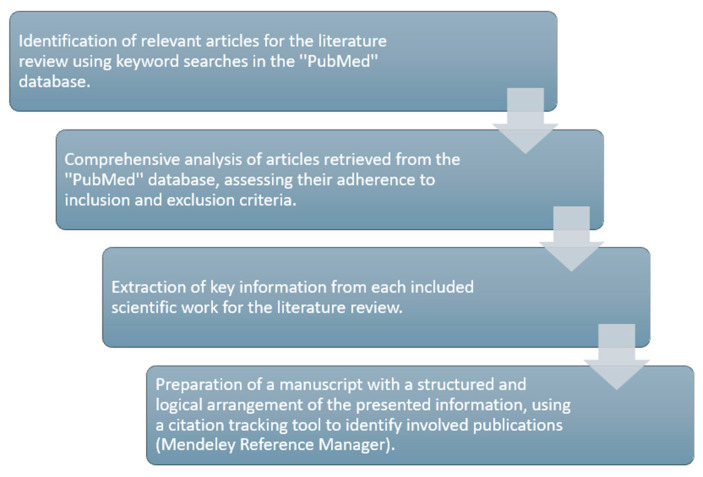
Schematic summary of the method used during preparation of the presented review of the literature.

From each article that was selected and included for review, we extracted the information that we considered valuable concerning the presented subject, according to each sub-section, to elaborate the present literature review. In total, 67 papers were included in the present manuscript, and citation tracking was used to identify the included publications (Mendeley Reference Manager) (Figure 1).

Each description of the intrinsic muscles of the foot was classified based on the classical clinical division. The following information was given for each muscle where possible: typical anatomy, morphological variations, clinical significance, and occurrence in ultrasound studies.

## 3. Discussion

### 3.1. Dorsal Aspect of the Foot

**Figure 2:**
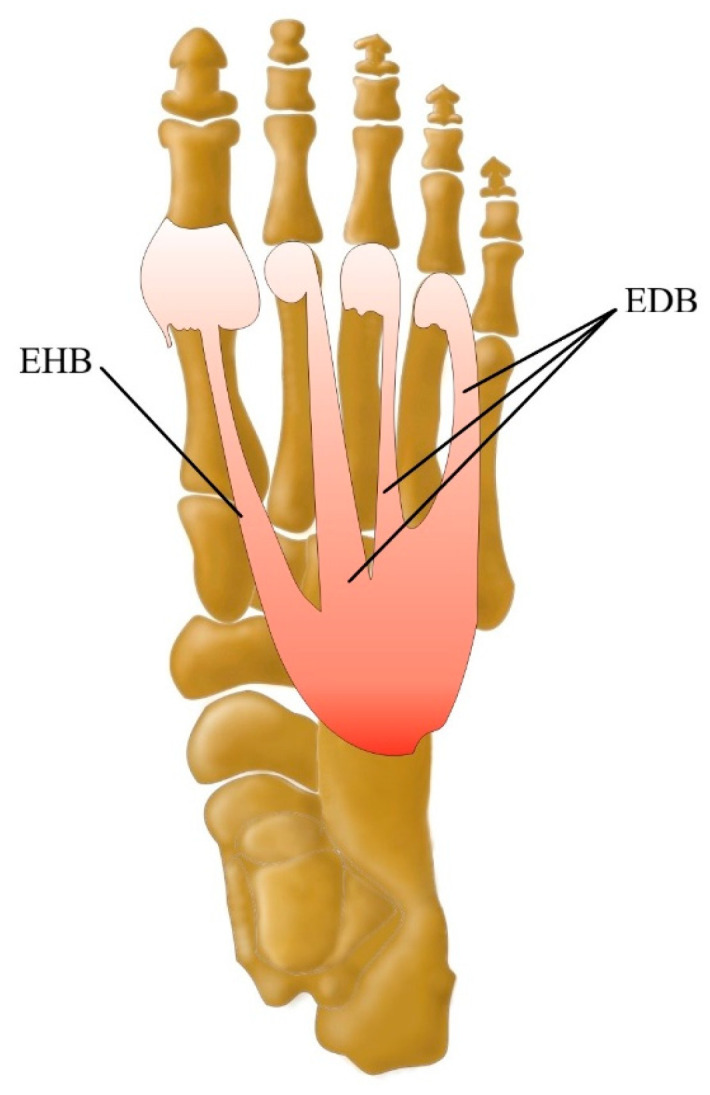
Dorsal muscles of the foot including the EHB extensor hallucis brevis muscle and the EDB extensor digitorum brevis muscle.

The dorsal aspect of the foot is composed of two muscles including the extensor digitorum brevis muscle (EDB) and the extensor hallucis brevis muscle (EHB) (Figure 2).

#### 3.1.1. Extensor Digitorum Brevis Muscle

The EDB is located on the dorsum of the foot. Most reports indicate that it originates from the superolateral surface of the calcaneus, interosseous talocalcaneal ligament, and inferior extensor retinaculum. It forms muscle bellies that merge into tendons and inserts onto the distal interphalangeal joints of the second, third, and fourth toes [17]. 

The EDB muscle demonstrates considerable variability with regard to the number of muscular heads and tendons, which vary from two to five [3]. Sirasangandla et al. [4] found four heads to predominate (90.9%) with some three-headed combinations (9.09%). A fifth belly to the fifth digit also was reported [18]. Interestingly, the number of bellies is associated with the insertion pattern (Table 1). 

In addition, the number of tendons also varies. Chaney et al. [19] reported the prevalence of dual tendons to be 3.8%, while Sirasangandla et al. [4] found multiple tendons to be present in 9.09% of the studied EDB muscles. 

EDB muscle tendons are currently used during surgical treatment of metatarsophalangeal joint degeneration caused by Freiberg’s disease, i.e., osteochondrosis of the lesser metatarsal head [20]. This would be a good use for additional EDB tendons, especially if they are easily accessible and of a suitable length [20]. Since the sacrifice of EDB tendons does not result in digital instability or deformity, they can be safely used during surgical procedures [20]. 

Additional heads of the EDB also could be clinically relevant, especially since the EDB is positioned superficially to the deep peroneal nerve. The presence of supernumerary heads could potentially lead to nerve compression and cause sensory disturbances of the dorsal surface area of the big toe and the second toe.

As the anatomical variation in the EDB muscle and its tendon may be clinically relevant, and as the muscle is easily visible during ultrasound examination with the use of the transverse plain, its variability merits particular attention [14]. Clinicians could consider US examination of the EDB when planning the surgical treatment of Freiberg’s disease or when identifying the cause of sensory disturbances of the dorsum of the foot. These examinations should also consider the variability in the muscle, especially with respect to the number of its heads and tendons. It is possible that an additional head of the EDB visualized on a US image may be confused with other hypoechoic and hyperechoic structures such as sarcoma or lipomas [21].

#### 3.1.2. Extensor Hallucis Brevis Muscle

The EHB originates together with the EDB from the superolateral surface of the calcaneus, the lateral talocalcaneal ligament, and the apex of the inferior extensor retinaculum and inserts into the dorsal surface of the base of the proximal phalanx of the first toe [17]. Because of its morphological presentation, the EHB is usually considered as the medial part of the EDB [17]. 

A variation of the extensor hallucis longus muscle (EHL), the extensor primi internodii hallucis muscle (EPIH), has been found to merge with the EHB [5]. The EPIH is an accessory tendinous slip that inserts distally to the base of the proximal phalanx of the hallux, distal/medial/directly to the EHB tendon distal attachment. The EPIH is characterized by its own muscular belly that originates from the EHL and traverses the metatarsophalangeal joint capsule; the EHB tendon is inserted into the EPIH tendon, merged, and inserted onto the base of the proximal phalanx of the hallux [5]. 

Patel et al. [22] also observed an interesting, bilateral variation connected with the EHB. An additional tendon was found to arise from the lateral aspect of the tibialis anterior muscle, an accessory tendon from the lateral surface of the EHL, and an accessory tendon from the superficial surface of the EHB that joined the distal end of the EHB muscle belly and ran superficially parallel to the main EHB tendon [22]. Interestingly, the additional EHL and EHB tendons merged, formed a common tendon, and inserted onto the dorsal aspect of the base of the proximal phalanx [22].

As the EHB is commonly used in transfer and tendinosis surgeries, EHB variations like EPIH-EHB tendinous communication or the additional tendons described by Patel et al. [22] may be of clinical significance. The EHB can be used for the correction of drop toe deformity [23], the correction of traumatic hallux varus [6,24], upper lip reconstruction [7], or the treatment of diabetic patients [25]. EHB transfer would be complicated in the case of EPIH-EHB union or where other tendons are joined. Unfortunately, these variations were not reported in previous ultrasound studies; however, their detection during pre-surgery imaging might be crucial when choosing EHB graft treatment. 

To visualize the musculoskeletal components of the dorsal aspect of the foot, it is recommended to use a linear high-frequency transducer, and the structures should be examined in the longitudinal and transverse planes [26]. Patients should be positioned supine with the ankle placed beyond the edge of the examination table to allow unimpeded active and passive movement at the ankle joint [26]. Additionally, dynamic evaluation should be routinely performed [26]. 

### 3.2. Plantar Aspect of the Foot

#### 3.2.1. Medial Plantar Muscles

**Figure 3:**
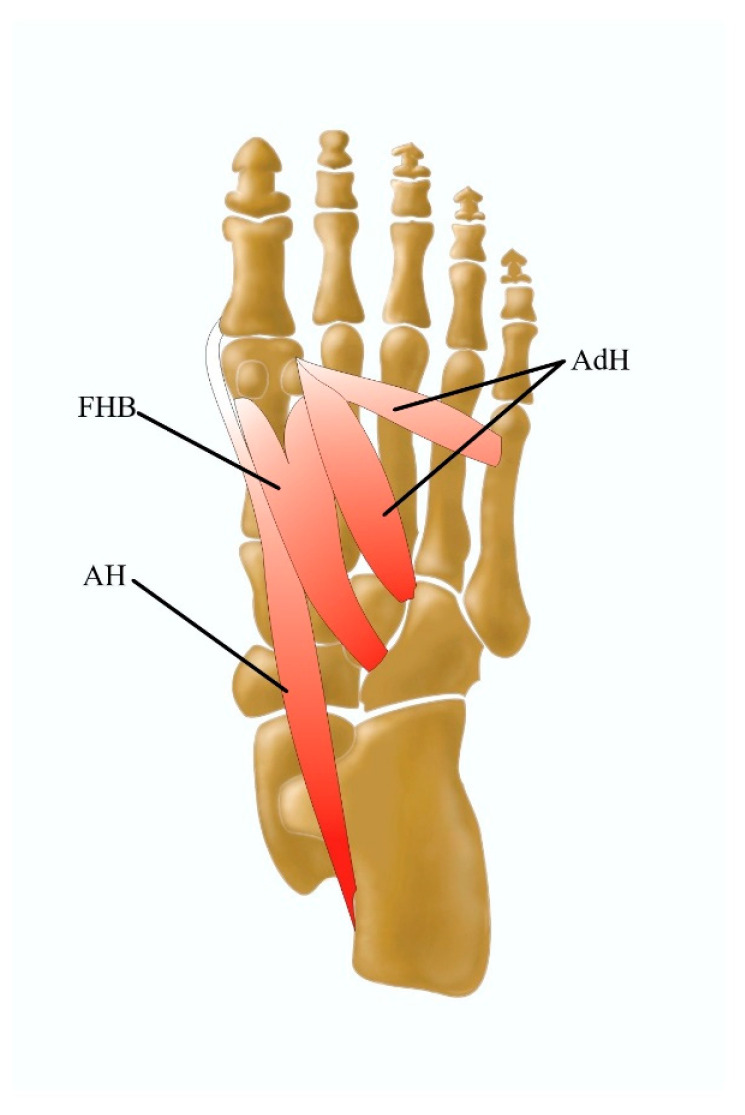
Medial plantar muscles including the AH abductor hallucis muscle, the AdH adductor hallucis muscle, and the FHB flexor hallucis brevis muscle.

The medial plantar muscular compartment of the foot consists of the abductor hallucis muscle (AH), the adductor hallucis muscle (AdH), and the flexor hallucis brevis muscle (FHB) (Figure 3).

#### 3.2.2. The Abductor Hallucis Muscle

The abductor hallucis (AH) is usually described as a muscle that arises from the calcaneal tuberosity, flexor retinaculum, plantar aponeurosis, and medial intermuscular septum [15]. The tendon is attached to the proximal phalanx of the hallux together with the medial tendon of the FHB [15]. 

**Figure 4:**
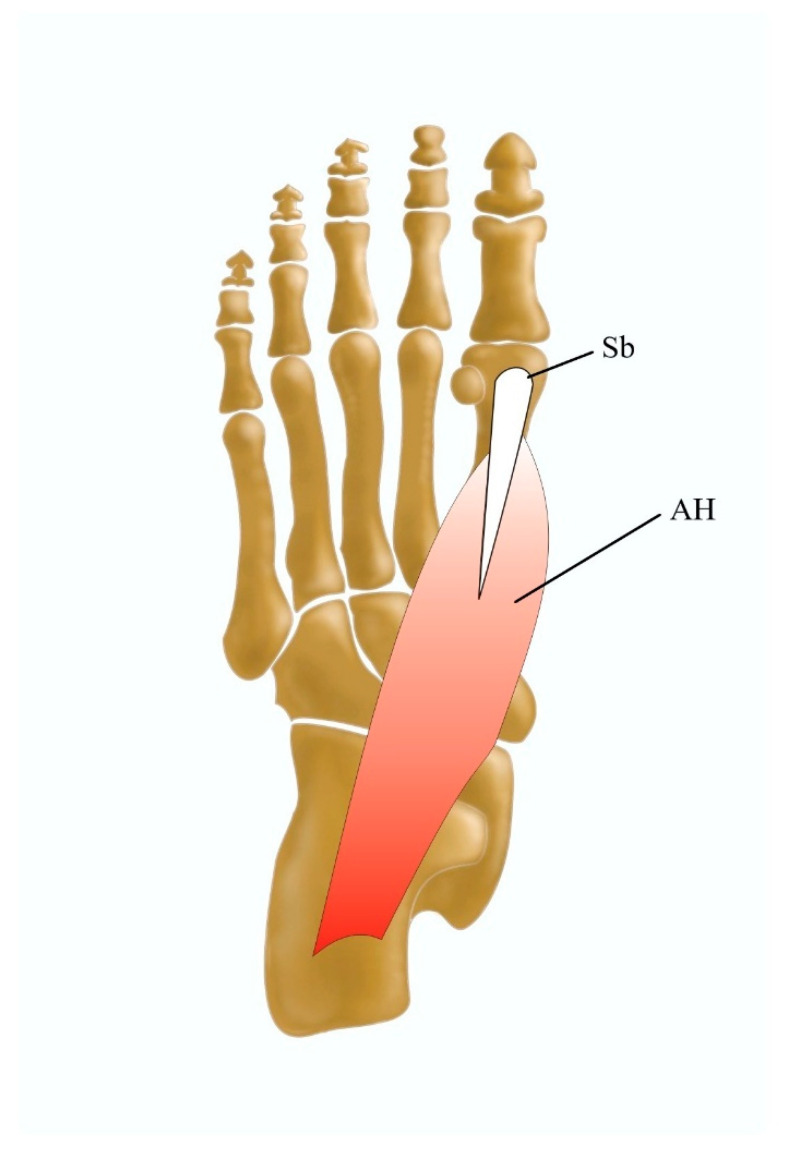
Morphological variation in the abductor hallucis muscle. AH, abductor hallucis muscle; Sb, medial sesamoid bone.

Even though the AH is not widely regarded as having a variable morphology, Agawany and Meguid [27] distinguish four categories of insertion (Table 2) (Figure 4).

Brenner [28] distinguishes three types of insertion (Table 3). 

The AH muscle is commonly used in clinical procedures since it is considered as unvaried and easily accessible surgically [8]. The AH is a frequent source of a local flap in reconstructive surgery of ankle and foot defects and as a free flap in reanimation of the cheek in fascial palsy [8]. The muscle is also applied in procedures that manage congenital deformities of the foot in pediatric surgery [27]. Moreover, the AH muscle may present an alternative in microvascular surgery since it can be used as a convenient source of the pedicled flap [27]. It can also be used as a reliable parameter for monitoring lower limb recovery after spinal cord injury [29]. Importantly, any kind of variance in the AH muscle could present a problem during surgery, particularly since its morphology is regarded as invariable [8].

However, Chittoria et al. [8] presented a case study in which the AH muscle demonstrated many tendinous attachments to the medial intermuscular septum; these arose from the central and tibial compartments of the plantar aponeurosis, the medial surface of the first metatarsal, and the muscular septum between the AH muscle and FHB. It demonstrated a higher tendon–muscle ratio than usual (1:1.76) and might not allow expected coverage if used as a flap [8]. Such unexpected variations could significantly extend operation time, which is a risk factor for possible infection. This is particularly important in patients with diabetes, who frequently undergo flap reconstructions [30].

An accessory AH has also been noted, although its occurrence is rather rare. Edwards et al. [31] describe such instances as a cause of tibial nerve entrapment. In addition, a case study identified an accessory AH originating from the fascia located superficially to the posterior tibial nerve, around 4 cm proximally to the medial malleolus [32]. The belly formed more than half a circle around the posterior tibial nerve, passed through the tarsal tunnel, and inserted into the main AH. The additional AH was found to be a cause of posterior tibial nerve entrapment during surgical treatment of the tarsal tunnel. Previously, the cause of the described entrapment was identified as a rheumatoid [32]. 

Nevertheless, the AH can be detected during ultrasound examination. If measured longitudinally in the location corresponding to the muscle belly, it is easy to achieve a clear image, with a large cross-sectional area of muscle tissue [14]. Such examination should be considered as part of pre-surgery preparation for procedures involving any kind of flap derived from the AH muscle; the images could reduce the likelihood of discovering an unexpected morphological variant during surgery. An imaging study should also be undertaken in the event of tarsal tunnel since it can be caused by ganglion cysts, bone spurs, varicose veins, injuries such as ankle sprains or fractures, lipomas, or other tumors located near the tibial nerve and the accessory/variable muscles [16,31,33]. However, as reports of an accessory AH are rare, misdiagnosis is still possible, even when US imaging is undertaken. Many clinicians might not be aware of such muscles and can misinterpret the US image or confuse the accessory AH with other causes of posterior tibial nerve entrapment, such as cysts or soft tissue tumors [16,31,34].

#### 3.2.3. The Adductor Hallucis Muscle

The AdH is composed of transverse and oblique heads. The transverse head originates from the plantar ligaments of the metatarsophalangeal joints, and the oblique head from the base of the second, third, and fourth metatarsals [17]. Both heads fuse before the insertion point at the lateral side of the base of the proximal phalanx of the first digit [17].

The AdH muscle presents substantial morphological variability regarding both of its heads; however, a greater range of variations has been observed in the transverse head [3]. A comprehensive classification of the AdH that accurately shows its remarkable variability was presented by Arakawa et al. [35]; the classification divided the oblique head into four types, as presented in Table 4.

Arakawa et al. [35] present a threefold classification of the transverse head (Table 5). 

The distal attachment also varies from 4 to 16 mm in width [36]. According to Sarrafian [37], the oblique head is composed of three components, each with unique attachments to the fibular sesamoid and an additional attachment of the lateral component, which also inserts onto the base of the proximal phalanx. Unlike the oblique head, the transverse head contains fibers that pass through other head fibers at the sesamoid level and that terminate on the flexor hallucis longus muscle (FHL) sheath [37]. Additionally, both heads conjoin and form a tendon with an insertion onto the lateral aspect of the base of the proximal phalanx [37]. The AdH muscle also occasionally presents a cord-like conjoined insertion of the oblique head and FHB [36].

The AdH muscle can also contribute to hallux valgus deformity by pulling on the lateral aspect of the first digit. In 1928, McBride [38] proposed that hallux valgus deformity was caused by laterally directed traction resulting from the adductor tendon inserting into the lateral sesamoid and that treatment should aim to reduce the first intermetatarsal angle by plantar–lateral soft tissue contracture liberation, exostectomy, fibular sesamoidectomy, and transposition of the adductor hallucis tendon [39]. Although many adjustments and modifications have been made to the procedure over the years, variations in AdH morphology could complicate any of them. For example, both the FHB and AdH tendinous fibers are present at the proximal phalanx base; if they are conjoined, the release of both tendons from the proximal phalanx could cause a reversal of muscle imbalance, resulting in hallux varus [40].

In addition, the variability in AdH insertion might complicate other procedures involving the resection of tendons inserted onto the base of the proximal phalanx of the hallux. These include metatarsophalangeal arthroplasty with/without tissue interposition, total implant arthroplasty or hemi-implant arthroplasty, or even amputation through the base of the proximal phalanx of the hallux [41].

Since the AdH muscle presents quite a significant variability and might surprise clinicians during many surgical procedures, some kind of visualization of this muscle during pre-operative preparations is advised. However, the first dorsal interosseus muscle, first lumbrical muscle, and AdH are present together in the ultrasound image, and it is very difficult if not impossible to obtain a clear indication of their edges, let alone their exact morphology. This may in fact complicate the planning resulting from the misidentification of such variants as other muscle or soft-tissue structures.

#### 3.2.4. The Flexor Hallucis Brevis Muscle

The FHB is composed of a lateral head arising from the medial part of the plantar surface of the cuboid bone and the lateral cuneiform bone [17], and a medial head arising from the lateral division of the tibialis posterior muscle tendon and the middle band of the medial intermuscular septum [17]. The divided bellies run anteriorly and medially towards the great toe and insert onto the base of the proximal phalanx of the hallux [17]. 

Few documented morphological variations in the FHB have been noted, and it can be considered as rather invariable. According to Le Double [42], the lateral head might be merged from the oblique head and the medial FHB head may unite with the AH tendon. Additional tendinous slips also might be present. Also, the lateral FHB head might produce a slight tendinous slip inserted onto the proximal phalanx of the second toe, and the medial head may produce a tendinous slip onto the medial cuneiform bone [42]. More recently, Young et al. [43] presented a case of congenital absence of tibial sesamoids connected with an alternate FHB tendon insertion. The authors noted a bilateral variation: the right foot had a bifurcation of the FHB with one slip blending into the AH tendon and capsule and the other slip traversing laterally and attaching to the lateral FHB tendon and fibular sesamoid [43]. On the left foot, however, the entire FHB traversed laterally and attached onto the fibular sesamoid and lateral FHB [43]. 

Recently, Masadeh et al. [44] reported the use of a distally based, reversed medial hemi-FHB muscle flap in the treatment of forefoot ulcerations in diabetic patients. The use of local flaps in this condition is beneficial, as they allow for the maintenance of the specialized anatomy while also providing rapid soft tissue coverage of the deep structures [25,45]. The local muscle flaps are limited by their size and arc of rotation, limiting their use to small, nearby defects. This can be overcome by using the muscle in a distal, or reverse way that allows the bulk of the muscle origin to fill the defect [44]. Masadeh et al. [44] described a method in which the flap is harvested from the medial head of the FHB; in such cases, it is vital to preserve the integrity of the first plantar metatarsal artery, as it is in close proximity. 

The reverse FHB muscle flap may be an excellent primary option for both free tissue transfer and ablative surgery in the management of soft tissue deficits of the distal first ray; however, the graft procedure might be technically difficult [44]. It cannot be ruled out that anatomical variations in the FHB such as additional tendinous slips could interfere with the described procedure. This might be a particular inconvenience when maintaining the continuity of the first metatarsal artery since the medial head of the FHB has been reported to have additional tendinous slips. It is unclear whether such variations could be visible in imaging studies, although further studies on this subject are required. 

In order to visualize the muscles that compose the medial plantar muscular compartment, the lateral border of the stabilized foot should be positioned with a neutral ankle position. The plantar aspect of the foot should be faced toward the floor to allow the linear transducer access to both the medial and plantar aspects of the foot [46]. 

### 3.3. Central Plantar Muscles

**Figure 5:**
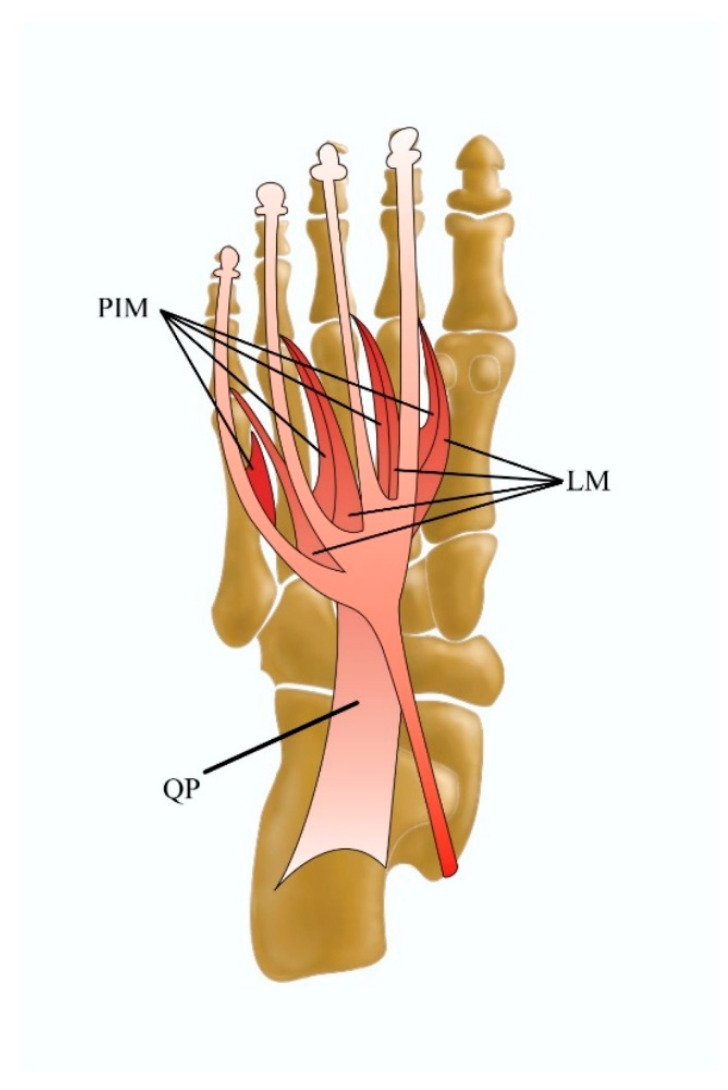
Central plantar muscles of the foot including the QP quadratus plantae muscle, the LM lumbrical muscles, and the PIM plantar interossei muscles.

The central muscular compartment of the plantar foot aspect is composed of the flexor digitorum brevis (FDB), quadratus plantae (QP), lumbrical muscles of the foot, and plantar interossei muscles (Figure 5). Even though a few variations of the interosseus muscles were previously reported [3,47,48], few ultrasound studies have discussed their clinical significance and occurrence. 

#### 3.3.1. The Flexor Digitorum Brevis Muscle

The flexor digitorum brevis (FDB) is a part of the first plantar layer of muscles contributing to plantar flexion of the foot [49]. It classically arises as a narrow tendon from the medial process of the calcaneus, the intermuscular septum, and the plantar aponeurosis. From here, it forms a muscular belly and divides into four tendons for the four lateral toes; the belly divides into two slips at the base of the proximal phalanges and attaches to both sides of the intermediate phalanx [50].

**Figure 6:**
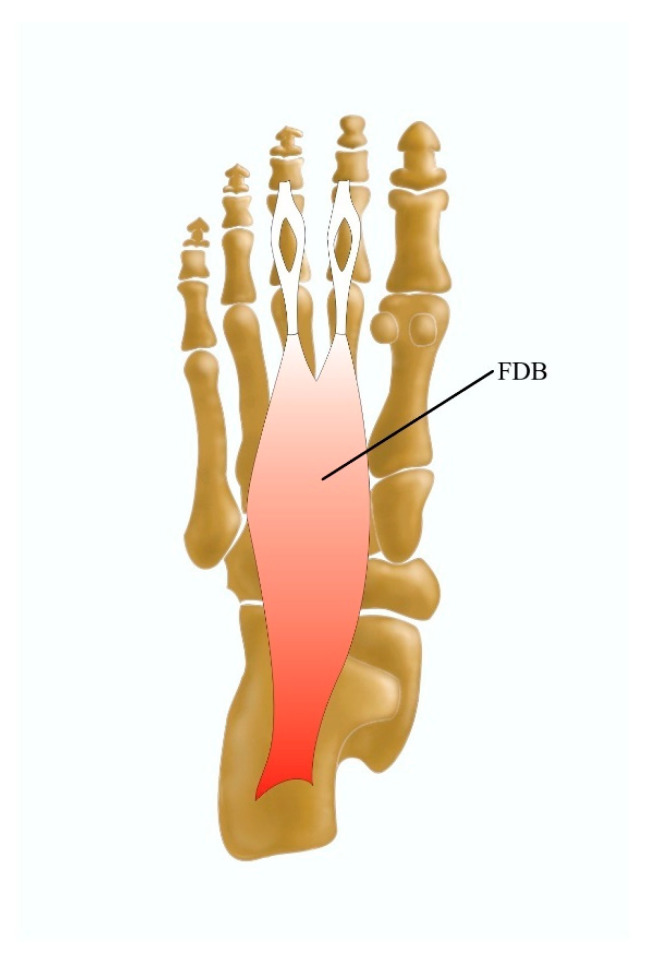
Morphological variation of the flexor digitorum brevis muscle and FDB flexor digitorum brevis muscle.

The FDB was reported to occur with 63% variable morphology [3]. The most commonly described variation is the lack of a muscle belly or tendon to the fifth toe, found in 2% to 100% of dissected cadavers [3,51]; these can sometimes be accompanied by the absence of the tendon to the fourth digit [52] (Figure 6). Slips for the fifth digit might be so small and slender that they insert onto the plantar fascia before reaching the actual fifth toe structure [53]. The flexor digitorum longus muscle (FDL), QP, or intermuscular septum might contribute slips to the lateral portions of the FDB or slips to the fifth toe when the tendon from the FDB is absent [3].

Interestingly, intertendinous connections were also noted between the FDB and FDL on both cadaveric dissections and dynamic ultrasound examinations; these connections continued from the ventral surface of the tendinous chiasm of the FDB and ended at the dorsal part of the FDL [54]. Such connections might be clinically significant in the event of rupture of one of the conjoined tendons or during percutaneous tenotomy for the treatment of claw toes since such procedures on the FDL reinforce the action of the FDB on the middle phalanx [55]. Furthermore, as mentioned before, the fourth tendon might be absent [49,52]. According to Bernhard et al. [49], 48% of dissected feet lacked a fourth tendon. Such absence might be clinically relevant in surgical or mechanical treatment since it alters the biomechanics of the fifth toe [49]. Additionally, the FDB itself is frequently used in reconstructive surgeries, such as those associated with the heel pad, where the FDB musculocutaneous flap is transferred; in such cases, knowledge of an absent tendon might be useful during pre-surgery planning [49,50].

The FDB occurs as quite a variable muscle. It can surprise clinicians during percutaneous tenotomy for the treatment of claw toes, heel pad reconstruction, or any kind of reconstructive surgery involving the FDB muscle. In such cases, pre-operative ultrasound imaging could be performed, especially since the FDB is easily visible when the probe is positioned perpendicular to a line from the medial calcaneal tubercle to the third toe [56].

#### 3.3.2. Quadratus Plantae

The QP muscle was first presented as the moles carnee in 1560 by Sylvius [57]. Since then, this muscle has been referred to as the caro plantae pedis quadrata or caro quadrata, but it is now known as the flexor digitorum accessorius or the QP [58]. Typically, this muscle originates from two heads separated by the long plantar ligament, i.e., the lateral head, from the lateral site of the plantar surface of the calcaneus and long plantar ligament, and a medial head, from the medial surface of the calcaneus [59]. Both of these heads attach to the FDL tendons [59]. Interestingly, the QP muscle does not have a corresponding muscle in the hand, and its medial head is unique to humans [58].

The QP muscle demonstrates considerable variation with regard to its site of origin and insertion. Pretterklieber [58] reported a two-headed QP in 57% of the studied feet, a one-headed variant in 34%, and a three-headed [60] variant in 9%. No examples of complete absence were noted. The medial head originates from the plantar calcaneal surface and medial process of the calcaneal tuberosity; it is also rarely associated with the plantar calcaneonavicular ligament [58,61]. The lateral head derives from the lateral process of the calcaneal tuberosity, plantar or lateral surface of the calcaneus [58,60,61]. The additional middle head, if present, usually arises from the medial or plantar surface of the calcaneus or from the lateral process of the calcaneal tuberosity [58,60]. 

Such incredible variability could be a potential cause of flatfoot or other deformities; it is also possible that differences in the number of QP heads or their thickness could predetermine specific individuals for different types of sports [58]. Additionally, knowledge of QP variability might be crucial in the treatment of abscesses in the diabetic foot or clubfoot [58].

Haratizadeh et al. [59] presented an interesting case study in which the QP muscle was found to have a tendinous site of insertion divided into three tendons that inserted onto the second, third, and fourth tendons of the FDL; these demonstrated comparable thickness to the FDL tendons. Additionally, a tendinous slip onto the FHL tendon was also noted [59]. The QP can be easily injured following a fracture of the calcaneus, and since the FHL and FDL tendons are common sources of grafts when treating posterior tibial tendon dysfunction or Achilles tendon rupture [59], the occurrence of such tendinous slips may present a problem. 

Therefore, some kind of pre-operative imaging examination is strongly recommended before these procedures. According to Mickle et al. [62], the QP muscle is easy to detect during ultrasound imaging. However, further US studies are needed, especially ones based on QP morphology and variability, as no such study has visualized additional slips. It can also be speculated that the third head could be confused with other soft tissue structures whose US image varies from hypoechoic to hyperechoic; however, further research is needed in this area.

#### 3.3.3. Lumbrical Muscles

The lumbrical muscles of the foot are sometimes referred to as accessory muscles of the FDL because of the fact that they originate directly from the FDL tendons. The first lumbrical muscle originates from the medial side of the first FDL tendon, i.e., the one intended for the second toe [17]. The remaining three lumbricals have two origin sites arising from the facing surfaces of two adjacent tendons of the FDL [17]. From its origin, each lumbrical muscle passes anteriorly to the base of the proximal phalanges of the second to fifth digits and their extensor expansions [17]. 

The origins of the lumbrical muscles vary considerably, i.e., from the tibialis posterior muscle tendon, the FHL tendon, or even from the FHB [3]. Interestingly, Hur et al. [63] noted that the muscle may originate as two muscle bellies from the FDL tendon and the tendinous slip of the FHL and then merge to form the muscle belly of the first lumbrical [63]. The authors classify the origin into six types (Table 6).

Interestingly, Oukouchi et al. [64] reported that the lumbrical muscles might produce accessory slips that form additional insertions (Table 7). 

Interestingly, Oukouchi et al. [64] also observed a variation, where the third and fourth lumbrical muscles were terminated by sending two tendons. The third lumbrical was inserted onto the base of the proximal phalanx on the second and third toe, while the fourth lumbrical was inserted onto the base of the proximal phalanx of the third and fourth toe [64]. 

Additionally, one/more lumbrical muscles might be absent. Chaney et al. [19] found one or more lumbricals to be absent in 45.5% of dissected feet and Schmidt et al. [65] in 8%.

Further studies are needed to determine the clinical significance of the morphological variations in the lumbrical muscles and their visibility in imaging studies. It is possible that the presence of additional tendinous slips, unusual arrangements of the distal insertion, or variations in the first lumbrical muscle origin [63] might complicate surgical procedures on the foot and ankle or disturb rehabilitation programs. In addition, Severinsen et al. [66] found that when the first lumbrical muscle and the AdH are present together in an ultrasound image, it is very difficult to indicate their outlines clearly, let alone their exact morphology. Nevertheless, further anatomical studies, biomechanics research, and radiological visualization attempts may resolve this. 

### 3.4. Lateral Plantar Muscles

**Figure 7:**
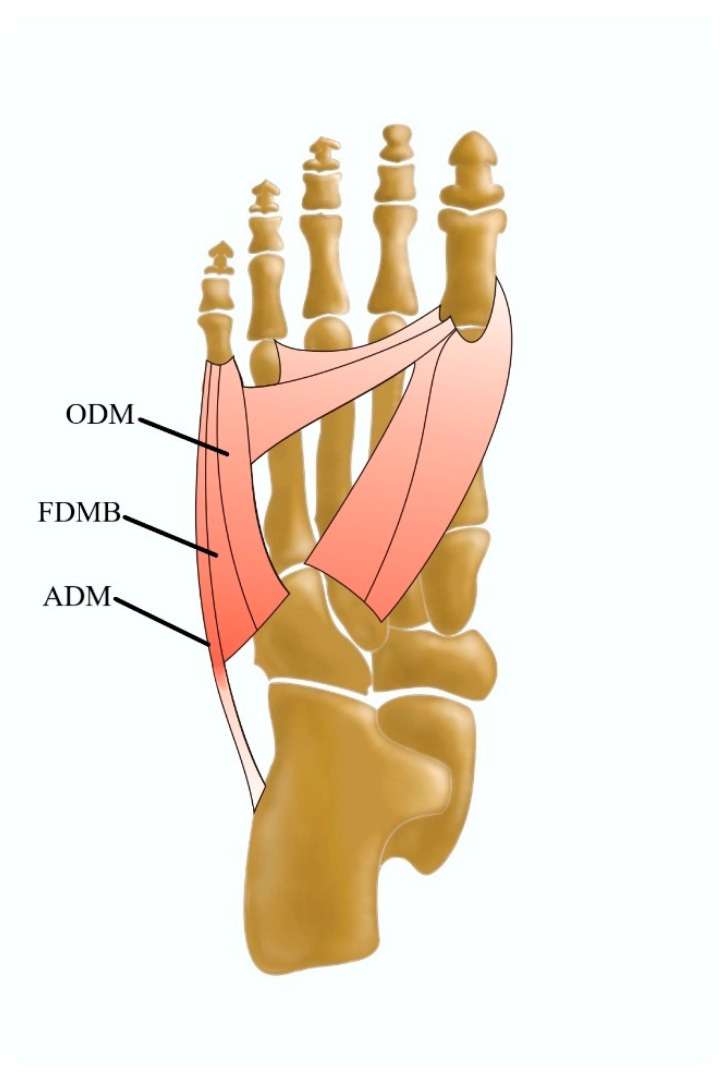
Lateral plantar muscles including the ADM abductor digiti minimi muscle, the FDMB flexor digiti minimi brevis muscle, and the ODM opponens digiti minimi muscle.

The lateral plantar muscular compartment includes the abductor digiti minimi muscle (ADM) and flexor digiti minimi brevis muscle [17]. Sometimes a third muscle, a slip from the flexor digiti minimi brevis muscle, that inserts onto the lateral border of the fifth metatarsal shaft/head, may form a separate muscular belly called the opponens digiti minimi [3] (Figure 7). A few other variations of the latter were reported, such as intermuscular connections with the ADM or its variations [3]; however, current information on the morphological variations, clinical significance, and US imaging of the flexor digiti minimi brevis and opponens digiti minimi is insufficient. 

#### The Abductor Digiti Minimi Muscle

The ADM is a muscle located in the lateral compartment of the plantar region that arises from the calcaneal tuberosity, plantar aponeurosis, and intermuscular septum and inserts onto the base of the proximal phalanx of the fifth metatarsal bone and fifth digit. 

**Figure 8:**
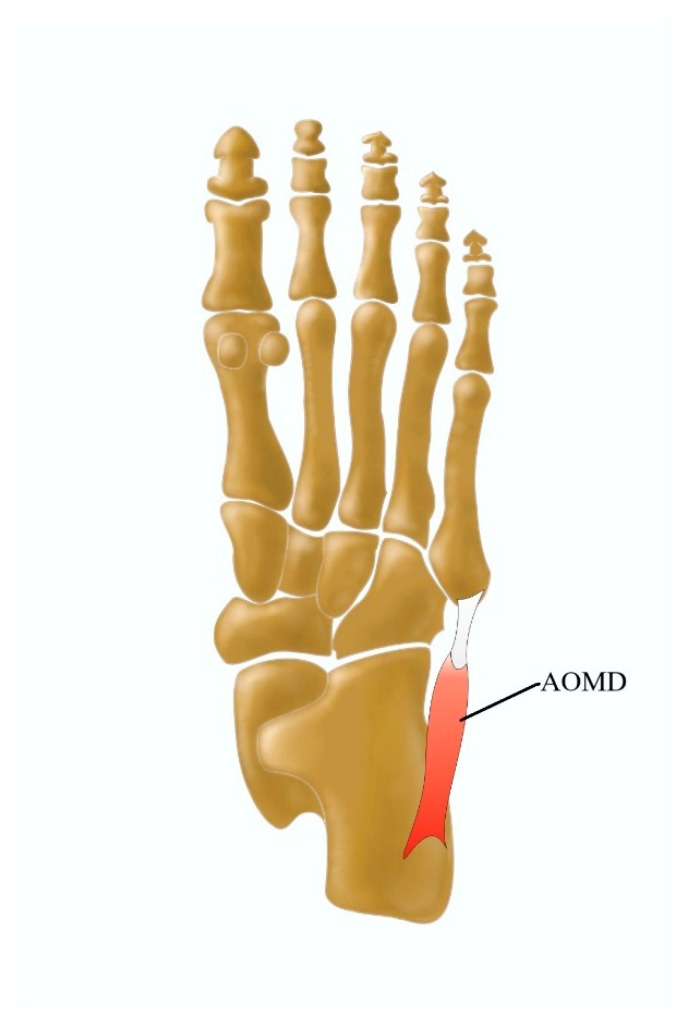
Morphological variation of the abductor digiti minimi muscle and AOMD abductor osi metatarsi digit minimi.

The morphological variability in the ADM manifests itself mostly in additional muscular components that are located close to the muscle or that arise directly from it [3]. The abductor osi metatarsi digit minimi is a fusiform-shaped muscular slip from the abductor digiti minimi with the site of insertion onto the styloid process of the fifth metatarsal bone [3,18] (Figure 8), with a prevalence of 43% during anatomical dissections carried out by Le Double [42].

Another variation of the ADM is the abductor accessories digiti minimi muscle, which is positioned just as abductor ossi metatarsi digiti minimi; however, it has its own origin from the posterolateral tuberosity of the calcaneus [3]. The ADM can also be considered as a two-headed muscle. Its superficial head forms the lateral margin of the foot, whereas the deep head is located laterally to the FDB and medially to the AH [67].

Its clinical significance is associated with the occurrence of subcalcaneal pain. The presence of two heads arising from both the medial and lateral processes of the calcaneal tuberosity causes the subdivision of the ADM nervous branch into two minor branches—posterior and anterior [67]. In such a situation, the posterior branch is positioned closely to the calcaneal tuberosity, which can contribute to compression or stretching of the nervous branch and cause subcalcaneal pain in the case of calcaneal spur occurrence [67].

The evaluation and exclusion of potential causes of subcalcaneal pain, such as fasciitis, trauma, vascular spasm, peroneal fibrosis, or the presence of a painful calcaneal spur are crucial stages in the process of diagnosis. Furthermore, compression of the posterior nervous branch caused by the presence of two heads of the ADM may be responsible, which might surprise a clinician during an examination. In such cases, a simple ultrasound examination of the ADM might be useful. To locate this muscle, the probe must be placed on the lateral calcaneal tuberosity, aiming directly toward the fifth metatarsal tuberosity [62].

## 4. Conclusions

The intrinsic muscles of the foot, like those of other regions of the human body, present extensive morphological variability and most are also easily detected during ultrasound examination. The variability in the intrinsic muscles of the foot manifests not only as a cause of numerous medical conditions but also as a source of material in numerous surgical procedures. The EDB can be used in the treatment of Fraiberg’s disease, the EHB is used in transfer and tendinous surgeries, the AH may be an excellent flap source in the reconstruction of the foot region or during the treatment of facial palsy, the AdH may interfere with hallux valgus or metatarsophalangeal arthroplasty, and many more. Any kind of morphological variability in the foot muscles of the foot region may cause complications during the operation and post-operation period. Therefore, it is important to properly visualize those structures during pre-operation preparations. 

It is crucial that, unlike other muscular regions of the body, muscles of the foot are relatively poorly understood, especially regarding imaging reports. To understand their clinical significance fully, further studies are required. 

## 5. Limitations

The presented review of the literature was written based on articles published only in one scientific database—“PubMed”.

## Figures and Tables

**Table 1:** Typical patterns of EDB insertion based on the number of muscle bellies.

Number of EDB Muscle Bellies	Typical Site of Insertion
2	first and fourth toes
	second and third toes
	first and fifth toes
3	first, second, and third toes
	second, third, and fourth toes
4	first, second, third, and fourth toe

**Table 2:** AH classification according to Agawany and Meguid [27].

Type	Description of Insertion	Occurrence
I	single tendinous insertion onto the base of the proximal phalanx of the first toe	46.7%
II	tendinous insertion onto the base of the proximal phalanx of the first toe with a slip onto the medial sesamoid bone	33.3%
III	single insertion onto the medial sesamoid bone	6.7%
IV	superficial tendinous slip onto the proximal phalanx and a deep slip onto the metatarsophalangeal joint capsule of the first toe	13.3%

**Table 3:** AH classification by Brenner [28].

Type	Description of Insertion	Occurrence
A	single tendon inserted onto the proximal phalanx of the first toe	38.5%
B	two insertion sites including the medial sesamoid bone and medial sesamoid ligament	59.6%
C	single insertion onto medial sesamoid bone	1.8%

**Table 4:** Classification of the AdH oblique head by Arakawa et al. [35].

Type	Description		Occurrence
A	Narrow type with three subtypes		47%
	Subtype 1	origin from the fibrous sheath of the peroneus longus muscle tendon, long plantar ligament, and the base of the second, third, and fourth metatarsals and the lateral cuneiform bone	
	Subtype 2	all sites of origin comparable with those presented in subtype 1; however, no origin from the long plantar ligament	
	Subtype 3	all sites of origin comparable with those presented in subtype 1; however, no origin from the lateral cuneiform bone	
B	Lateral type; origin from the fibrous sheath of the peroneus longus muscle tendon, long plantar ligament, and the base of the second, third, and fourth metatarsals, the lateral cuneiform bone, and additionally from the base of the fifth metatarsal bone		33%
C	Wide type; compound origin: laterally from the fifth metatarsal bone, medially from the medial intermuscular septum/tibialis posterior tendon/plantar tarsometatarsal ligament/peroneus longus tendon, and classically from the fibrous sheath of the peroneus longus muscle tendon, the long plantar ligament, and the base of the second, third, and fourth metatarsals, and the lateral cuneiform bone		9%
D	Medial type; origin from the divided tendon of the tibialis posterior/medial intermuscular septum/tarsometatarsal ligament/peroneus longus tendon and from the fibrous sheath of the peroneus longus muscle tendon, the long plantar ligament, and the base of the second, third, and fourth metatarsals and the lateral cuneiform bone		11%

**Table 5:** Classification of the AdH transverse head according to Arakawa et al. [35].

Type	Description	Occurrence
A	Narrow type; origin from the third and fourth metatarsophalangeal joint capsules and from the deep transverse metatarsal ligaments	40%
B	Lateral type; origin from the third, fourth, and fifth metatarsophalangeal joint capsules and from the deep transverse metatarsal ligament	30%
C	Wide type; origin from the aponeurosis between the third plantar interosseus muscle and fourth dorsal interosseus muscle and from the third and fourth (sometimes also the fifth) metatarsophalangeal joint capsules	30%

**Table 6:** Types of first lumbrical muscle origin by Hur et al. [63].

Type	Description	Occurrence
1	two similarly sized muscle bellies from both the tendon of the FDL and the tendinous slip of the FHL for the second toe	37.9%
2	two-thirds of the muscle arose from the FDL tendon for the second toe, one-third of the first lumbrical arose from the tendinous slip of the FHL for the second toe	30.3%
3	origin solely from the FHL tendon for the second toe	15.2%
4	origin from the tendon of the FDL for the second toe with a few muscle fibers arising from the tendinous slip of the FHL for the second toe	12.1%
5	one-third of the muscle arises from the tendon of the FDL for the second toe and two-thirds from the tendinous slip of the FHL for the second toe	3%
6	origin solely from the tendon of the FDL for the second toe	1.5%

**Table 7:** Accessory lumbrical muscle slips observed by Oukouchi et al. [64].

Accessory Slip Origin	Accessory Slip Insertion	Occurrence
The first lumbrical muscle	The base of the second proximal phalanx	12%
The second lumbrical muscle	The base of the third proximal phalanx	8%
The third lumbrical muscle	The base of the fourth proximal phalanx	12%
The fourth lumbrical muscle	The base of the fifth proximal phalanx	16%

## Data Availability

Please contact the authors for data requests (Łukasz Olewnik, PhD—email address: lukasz.olewnik@umed.lodz.pl).
